# A second monoclinic polymorph of (*E*)-phen­yl(pyridin-2-yl)methanone oxime

**DOI:** 10.1107/S1600536813002377

**Published:** 2013-01-31

**Authors:** Monserrath I. Rodríguez-Mora, Reyna Reyes-Martínez, Marcos Flores-Alamo, Juventino J. García, David Morales-Morales

**Affiliations:** a Instituto de Química, Universidad Nacional Autónoma de México, Circuito exterior, Ciudad Universitaria, México, DF, 04510, Mexico; b Facultad de Química, Universidad Autónoma de México, México D. F., 04510, Mexico

## Abstract

The title compound, C_12_H_10_N_2_O, a second monoclinic poly­morph of (*E*)-phen­yl(pyridin-2-yl)methanone oxime crystallizes in the space group *P*2_1_/*n* (*Z* = 4). The previously reported polymorph [Taga *et al.* (1990 ▶). *Acta Cryst*. C**46**, 2241–2243] occurs in the space group *C*2/*c* (*Z* = 8). In the crystal, pairs of bifurcated O—H⋯(N,O) hydrogen bonds link the mol­ecules into inversion dimers. The dimers are linked by C—H⋯π inter­actions, forming a linear arrangement. The dihedral angle between the pyridine and phenyl rings is 67.70 (8)°.

## Related literature

For properties of oximes, see: Custot *et al.* (1996 ▶); Turner & Ciufolini (2011 ▶); Abele *et al.* (2003 ▶). For the use of complexes of pyridyl oximes with a variety of transition metals in supra­molecular and materials chemistry, see: Shokrollahi *et al.* (2008 ▶); Martinez *et al.* (2008 ▶). For the previously reported polymorph, see: Taga *et al.* (1990 ▶).
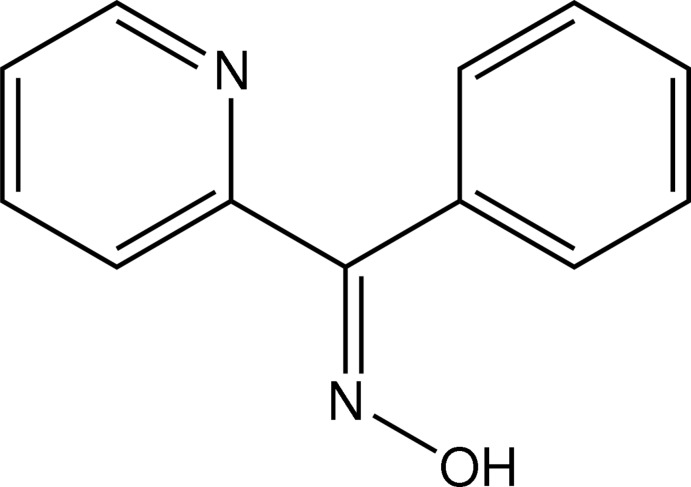


## Experimental

### 

#### Crystal data

C_12_H_10_N_2_O
*M* *_r_* = 198.22
Monoclinic, 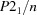

*a* = 5.6732 (4) Å
*b* = 23.257 (2) Å
*c* = 7.4516 (5) Å
β = 97.743 (7)°
*V* = 974.21 (13) Å^3^
*Z* = 4
Mo *K*α radiation
μ = 0.09 mm^−1^
*T* = 130 K
0.55 × 0.31 × 0.06 mm

#### Data collection

Agilent Xcalibur (Atlas, Gemini) diffractometer
Absorption correction: analytical [*CrysAlis PRO* (Agilent, 2011) ▶, based on expressions derived by Clark & Reid (1995 ▶)] *T*_min_ = 0.976, *T*_max_ = 0.995
4256 measured reflections
1918 independent reflections
1559 reflections with *I* > 2σ(*I*)
*R*_int_ = 0.023

#### Refinement

*R*[*F* ^2^ > 2σ(*F* ^2^)] = 0.042
*wR*(*F* ^2^) = 0.107
*S* = 1.04
1918 reflections
139 parameters
1 restraint
H atoms treated by a mixture of independent and constrained refinement
Δρ_max_ = 0.21 e Å^−3^
Δρ_min_ = −0.22 e Å^−3^

### 

Data collection: *CrysAlis PRO* (Agilent, 2011 ▶); cell refinement: *CrysAlis PRO*; data reduction: *CrysAlis PRO*; program(s) used to solve structure: *SHELXS97* (Sheldrick, 2008 ▶); program(s) used to refine structure: *SHELXL97* (Sheldrick, 2008 ▶); molecular graphics: *ORTEP-3 for Windows* (Farrugia, 2012 ▶) and *DIAMOND* (Brandenburg, 2006 ▶); software used to prepare material for publication: *PLATON* (Spek, 2009 ▶).

## Supplementary Material

Crystal structure: contains datablock(s) I, global. DOI: 10.1107/S1600536813002377/zj2100sup1.cif

Structure factors: contains datablock(s) I. DOI: 10.1107/S1600536813002377/zj2100Isup2.hkl

Additional supplementary materials:  crystallographic information; 3D view; checkCIF report

## Figures and Tables

**Table 1:** Hydrogen-bond geometry (Å, °) *Cg* is the centroid of the C1–C6 ring.

*D*—H⋯*A*	*D*—H	H⋯*A*	*D*⋯*A*	*D*—H⋯*A*
O1—H1*D*⋯N2^i^	0.88 (2)	1.93 (2)	2.7696 (17)	159 (2)
O1—H1*D*⋯O1^i^	0.88 (2)	2.61 (2)	3.225 (2)	127 (2)
C11—H11⋯*Cg* ^ii^	0.95	2.78	3.5453 (18)	139

Symmetry codes: (i) 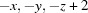; (ii) 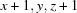.

**Fig. 1.**
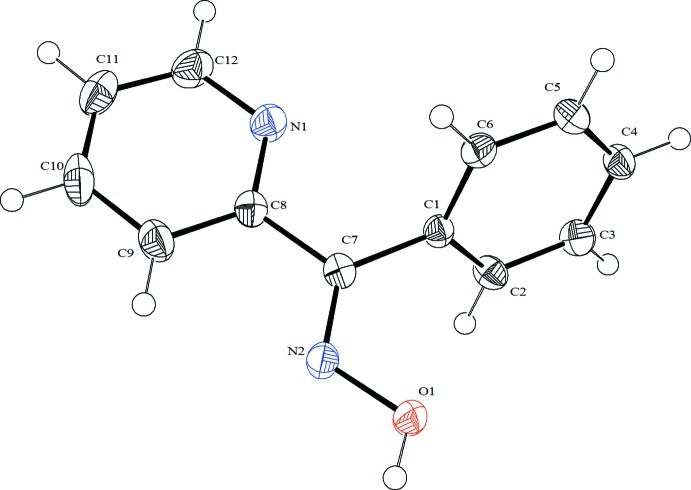
The molecular structure of the title compound with displacement ellipsoids at the 50% probability.

**Fig. 2.**
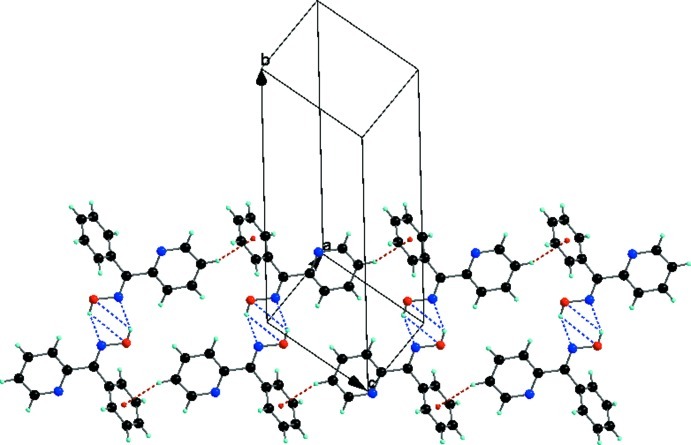
Intramolecular hydrogen-bonding interactions in the title compound, with hydrogen bonds shown as dashed lines.

## supplementary crystallographic information

### Comment

Oximes have been widely studied due to their biological and chemical properties, revealing, for instance, good activities as inhibitors of arginase (Custot *et al.*, 1996). Oximes are also preferred intermediates in the synthesis of compounds with biological activity (Turner *et al.*, 2011), for example, derivatives of Pyridine Oximes have been studied as antidotes against organophosphorus compounds poisoning, cytotoxic and antiviral agents,analgesic,antidepressants and tranquillizers (Abele *et al.*, 2003). Additionally, pyridil oximes are used in the preparation of complexes with a variety of transition metals, binding to metals in different forms most commonly as chelates or serving as bridge to metals, and the resulting species have been employed in supramolecular and materials chemistry (Shokrollahi *et al.*, 2008; Martinez *et al.*, 2008). Herein we report a second polymorph of (*E*)-Phenyl 2-Pyridyl Ketone Oxime (I) (figure 1).

In comparison, compound II described previously (Taga *et al.*, 1990), crystallized in a monoclinic (*C*2/*c*), while the title compound I crystallized in a space group *P*2_1_/*n*. In compound I the dihedral angle between the pyridine and phenyl rings is 67.70° (8), the orientation of the pyridine ring with a N(2)—C(7)—C(8)—N(1) angle of -174.04° is different from that of the polymorph previously reported of 37.5 (2)° (Taga *et al.*, 1990). The bonds distances C(7)—N(2) and N(2)—O(1) of the oxime group are close to those informed for their polymorph 1.283 (2) and 1.3391 (16) Å, respectively. In compound II was reported a bifurcated hydrogen bond between the OH group with the pyridine N atom and the oxime N atom. While in compound I the OH group forms a bifurcated hydrogen bond [O(1)—H(one-dimensional)···N(2) and O(1)—H(one-dimensional)···O(1)] with the oxime N atom and the hydroxyl O atom of the neighbouring oxime affording a centrosymmetric dimmer, these dimmers are kept together by C—H···π interactions (Figure 2).

### Experimental

The suitable crystal for X-ray study was obtained by slow evaporation of a solution of commercial (*E*)-Phenyl 2-Pyridyl Ketone Oxime in CH_2_Cl_2_.

### Refinement

H atoms attached to C atoms were placed in geometrically idealized positions, and refined as riding on their parent atoms, with C—H distances fixed to 0.95 (aromatic CH) with *U*_iso_ = 1.2 *U*_eq_(C). The hydroxyl H atom was located in a difference map and was refined with free coordinates and *U*_iso_(H) = 1.2 *U*_eq_(O).

### Crystal data

**Table:**

C_12_H_10_N_2_O	*F*(000) = 416
*M**_r_* = 198.22	*D*_x_ = 1.351 Mg m^−^^3^
Monoclinic, *P*2_1_/*n*	Mo *K*α radiation, λ = 0.71073 Å
*a* = 5.6732 (4) Å	Cell parameters from 1437 reflections
*b* = 23.257 (2) Å	θ = 3.5–26.0°
*c* = 7.4516 (5) Å	µ = 0.09 mm^−^^1^
β = 97.743 (7)°	*T* = 130 K
*V* = 974.21 (13) Å^3^	Lamina, pale pink
*Z* = 4	0.55 × 0.31 × 0.06 mm

### Data collection

**Table:**

Agilent Xcalibur (Atlas, Gemini) diffractometer	1918 independent reflections
Graphite monochromator	1559 reflections with *I* > 2σ(*I*)
Detector resolution: 10.4685 pixels mm^-1^	*R*_int_ = 0.023
ω scans	θ_max_ = 26.1°, θ_min_ = 3.5°
Absorption correction: analytical [*CrysAlis PRO* (Agilent, 2011), based on expressions derived by Clark & Reid (1995)]	*h* = −7→6
*T*_min_ = 0.976, *T*_max_ = 0.995	*k* = −28→25
4256 measured reflections	*l* = −9→6

### Refinement

**Table:**

Refinement on *F*^2^	Primary atom site location: structure-invariant direct methods
Least-squares matrix: full	Secondary atom site location: difference Fourier map
*R*[*F*^2^ > 2σ(*F*^2^)] = 0.042	Hydrogen site location: inferred from neighbouring sites
*wR*(*F*^2^) = 0.107	H atoms treated by a mixture of independent and constrained refinement
*S* = 1.04	*w* = 1/[σ^2^(*F*_o_^2^) + (0.0445*P*)^2^ + 0.3722*P*] where *P* = (*F*_o_^2^ + 2*F*_c_^2^)/3
1918 reflections	(Δ/σ)_max_ < 0.001
139 parameters	Δρ_max_ = 0.21 e Å^−^^3^
1 restraint	Δρ_min_ = −0.22 e Å^−^^3^

### Special details

**Table:**

Geometry. All s.u.'s (except the s.u. in the dihedral angle between two l.s. planes) are estimated using the full covariance matrix. The cell s.u.'s are taken into account individually in the estimation of s.u.'s in distances, angles and torsion angles; correlations between s.u.'s in cell parameters are only used when they are defined by crystal symmetry. An approximate (isotropic) treatment of cell s.u.'s is used for estimating s.u.'s involving l.s. planes.
Refinement. Refinement of *F*^2^ against ALL reflections. The weighted *R*-factor *wR* and goodness of fit *S* are based on *F*^2^, conventional *R*-factors *R* are based on *F*, with *F* set to zero for negative *F*^2^. The threshold expression of *F*^2^ > 2σ(*F*^2^) is used only for calculating *R*-factors(gt) *etc*. and is not relevant to the choice of reflections for refinement. *R*-factors based on *F*^2^ are statistically about twice as large as those based on *F*, and *R*- factors based on ALL data will be even larger.

### Fractional atomic coordinates and isotropic or equivalent isotropic displacement parameters (Å^2^)

**Table:**

	*x*	*y*	*z*	*U*_iso_*/*U*_eq_	
O1	−0.01894 (19)	0.05043 (5)	0.85027 (15)	0.0286 (3)	
N1	0.6746 (2)	0.13409 (6)	1.14331 (18)	0.0290 (3)	
N2	0.1685 (2)	0.04881 (5)	0.99337 (16)	0.0233 (3)	
C1	0.2991 (2)	0.13583 (6)	0.84972 (19)	0.0198 (3)	
C2	0.1085 (3)	0.17379 (7)	0.8370 (2)	0.0243 (4)	
H2	−0.0075	0.1701	0.917	0.029*	
C3	0.0870 (3)	0.21697 (7)	0.7082 (2)	0.0261 (4)	
H3	−0.0419	0.2433	0.7016	0.031*	
C4	0.2528 (3)	0.22192 (7)	0.5889 (2)	0.0263 (4)	
H4	0.2369	0.2513	0.4996	0.032*	
C5	0.4416 (3)	0.18395 (7)	0.6003 (2)	0.0279 (4)	
H5	0.5546	0.1871	0.5176	0.034*	
C6	0.4672 (3)	0.14138 (7)	0.7312 (2)	0.0249 (4)	
H6	0.5994	0.116	0.74	0.03*	
C7	0.3204 (2)	0.08948 (6)	0.98862 (19)	0.0201 (3)	
C8	0.5261 (3)	0.08876 (7)	1.13697 (19)	0.0219 (3)	
C9	0.5601 (3)	0.04407 (7)	1.2600 (2)	0.0308 (4)	
H9	0.4548	0.0121	1.2517	0.037*	
C10	0.7525 (3)	0.04717 (8)	1.3962 (2)	0.0382 (5)	
H10	0.7786	0.0173	1.4835	0.046*	
C11	0.9048 (3)	0.09311 (8)	1.4052 (2)	0.0346 (4)	
H11	1.0369	0.0958	1.4978	0.042*	
C12	0.8605 (3)	0.13487 (8)	1.2768 (2)	0.0335 (4)	
H12	0.9671	0.1665	1.2816	0.04*	
H1D	−0.097 (3)	0.0196 (8)	0.878 (3)	0.05*	

### Atomic displacement parameters (Å^2^)

**Table:**

	*U*^11^	*U*^22^	*U*^33^	*U*^12^	*U*^13^	*U*^23^
O1	0.0289 (6)	0.0258 (6)	0.0284 (6)	−0.0061 (5)	−0.0054 (5)	0.0065 (5)
N1	0.0306 (7)	0.0282 (8)	0.0266 (7)	−0.0043 (6)	−0.0018 (6)	−0.0015 (6)
N2	0.0250 (7)	0.0224 (7)	0.0215 (6)	0.0002 (5)	−0.0007 (5)	0.0012 (5)
C1	0.0225 (7)	0.0183 (8)	0.0179 (7)	−0.0019 (6)	0.0000 (6)	−0.0007 (6)
C2	0.0257 (8)	0.0235 (8)	0.0250 (8)	0.0008 (6)	0.0081 (6)	0.0014 (6)
C3	0.0271 (8)	0.0197 (8)	0.0312 (9)	0.0029 (6)	0.0033 (6)	0.0017 (7)
C4	0.0302 (8)	0.0242 (8)	0.0236 (8)	−0.0054 (7)	0.0004 (6)	0.0055 (6)
C5	0.0256 (8)	0.0350 (10)	0.0242 (8)	−0.0023 (7)	0.0069 (6)	0.0041 (7)
C6	0.0201 (7)	0.0286 (9)	0.0259 (8)	0.0023 (7)	0.0027 (6)	0.0020 (7)
C7	0.0233 (7)	0.0184 (7)	0.0193 (7)	0.0030 (6)	0.0050 (6)	−0.0007 (6)
C8	0.0243 (8)	0.0227 (8)	0.0192 (7)	0.0038 (6)	0.0049 (6)	−0.0009 (6)
C9	0.0350 (9)	0.0263 (9)	0.0316 (9)	0.0033 (7)	0.0062 (7)	0.0077 (7)
C10	0.0451 (10)	0.0414 (11)	0.0279 (9)	0.0161 (9)	0.0049 (8)	0.0138 (8)
C11	0.0298 (9)	0.0475 (11)	0.0246 (8)	0.0082 (8)	−0.0029 (7)	−0.0042 (8)
C12	0.0301 (9)	0.0377 (10)	0.0308 (9)	−0.0060 (8)	−0.0026 (7)	−0.0063 (8)

### Geometric parameters (Å, º)

**Table:**

O1—N2	1.4015 (15)	C4—H4	0.95
O1—H1D	0.882 (15)	C5—C6	1.384 (2)
N1—C8	1.346 (2)	C5—H5	0.95
N1—C12	1.349 (2)	C6—H6	0.95
N2—C7	1.2832 (19)	C7—C8	1.495 (2)
C1—C2	1.389 (2)	C8—C9	1.382 (2)
C1—C6	1.391 (2)	C9—C10	1.388 (2)
C1—C7	1.488 (2)	C9—H9	0.95
C2—C3	1.383 (2)	C10—C11	1.370 (3)
C2—H2	0.95	C10—H10	0.95
C3—C4	1.382 (2)	C11—C12	1.363 (3)
C3—H3	0.95	C11—H11	0.95
C4—C5	1.382 (2)	C12—H12	0.95
			
N2—O1—H1D	98.8 (13)	C1—C6—H6	120.1
C8—N1—C12	117.37 (14)	N2—C7—C1	124.14 (13)
C7—N2—O1	113.76 (12)	N2—C7—C8	115.55 (13)
C2—C1—C6	119.46 (14)	C1—C7—C8	120.29 (13)
C2—C1—C7	119.83 (13)	N1—C8—C9	122.33 (14)
C6—C1—C7	120.71 (13)	N1—C8—C7	116.07 (13)
C3—C2—C1	120.25 (14)	C9—C8—C7	121.60 (14)
C3—C2—H2	119.9	C8—C9—C10	118.12 (16)
C1—C2—H2	119.9	C8—C9—H9	120.9
C4—C3—C2	120.15 (14)	C10—C9—H9	120.9
C4—C3—H3	119.9	C11—C10—C9	120.34 (16)
C2—C3—H3	119.9	C11—C10—H10	119.8
C5—C4—C3	119.75 (15)	C9—C10—H10	119.8
C5—C4—H4	120.1	C12—C11—C10	117.75 (15)
C3—C4—H4	120.1	C12—C11—H11	121.1
C4—C5—C6	120.48 (15)	C10—C11—H11	121.1
C4—C5—H5	119.8	N1—C12—C11	124.08 (16)
C6—C5—H5	119.8	N1—C12—H12	118
C5—C6—C1	119.89 (14)	C11—C12—H12	118
C5—C6—H6	120.1		
			
C6—C1—C2—C3	−0.4 (2)	C6—C1—C7—C8	64.41 (19)
C7—C1—C2—C3	−179.93 (13)	C12—N1—C8—C9	−0.5 (2)
C1—C2—C3—C4	1.3 (2)	C12—N1—C8—C7	179.37 (14)
C2—C3—C4—C5	−0.7 (2)	N2—C7—C8—N1	−174.03 (13)
C3—C4—C5—C6	−0.6 (2)	C1—C7—C8—N1	4.9 (2)
C4—C5—C6—C1	1.5 (2)	N2—C7—C8—C9	5.8 (2)
C2—C1—C6—C5	−0.9 (2)	C1—C7—C8—C9	−175.19 (14)
C7—C1—C6—C5	178.56 (14)	N1—C8—C9—C10	1.2 (2)
O1—N2—C7—C1	2.2 (2)	C7—C8—C9—C10	−178.63 (15)
O1—N2—C7—C8	−178.89 (12)	C8—C9—C10—C11	−0.9 (3)
C2—C1—C7—N2	62.8 (2)	C9—C10—C11—C12	−0.1 (3)
C6—C1—C7—N2	−116.71 (17)	C8—N1—C12—C11	−0.6 (3)
C2—C1—C7—C8	−116.09 (16)	C10—C11—C12—N1	0.9 (3)

### Hydrogen-bond geometry (Å, º)

Cg is the centroid of the C1–C6 ring.

**Table:**

*D*—H···*A*	*D*—H	H···*A*	*D*···*A*	*D*—H···*A*
O1—H1*D*···N2^i^	0.88 (2)	1.93 (2)	2.7696 (17)	159 (2)
O1—H1*D*···O1^i^	0.88 (2)	2.61 (2)	3.225 (2)	127 (2)
C11—H11···*Cg*^ii^	0.95	2.78	3.5453 (18)	139

Symmetry codes: (i) −*x*, −*y*, −*z*+2; (ii) *x*+1, *y*, *z*+1.
